# Comparison of Redo percutaneous mitral valvuloplasty for mitral restenosis with first procedure for de novo mitral stenosis

**DOI:** 10.12669/pjms.38.6.5447

**Published:** 2022

**Authors:** Muhammad Ramzan, Muhammad Kashif Javed, Hafiz Muhammad Rizwan, Jahanzeb

**Affiliations:** 1 Dr. Muhammad Ramzan, MBBS, FCPS, Department of Cardiology Ch. Pervaiz Elahi Institute of Cardiology, Multan, Pakistan; 2 Dr. Muhammad Kashif Javed, MBBS, FCPS, Department of Cardiology Ch. Pervaiz Elahi Institute of Cardiology, Multan, Pakistan; 3 Dr. Hafiz Muhammad Rizwan, MBBS, FCPS, Department of Cardiology Ch. Pervaiz Elahi Institute of Cardiology, Multan, Pakistan; 4 Dr. Jahanzeb, MBBS, FCPS, Department of Cardiology Ch. Pervaiz Elahi Institute of Cardiology, Multan, Pakistan

**Keywords:** Mitral restenosis, Redo, Percutaneous mitral valvuloplasty, Mitral stenosis

## Abstract

**Objectives:**

To evaluate and compare the effects of redo percutaneous mitral valvuloplasty with initial percutaneous mitral valvuloplasty (PMV) in mitral restenosis (MR) and de novo mitral stenosis (MS) patients, respectively.

**Methods:**

A retrospective study was conducted at the cardiology department of Ch. Pervaiz Elahi Institute of Cardiology Multan for the period of one year from 6^th^ July 2020 to 6^th^ July 2021. A total of 50 patients were recruited in the study. Out of them, 20 de novo MS patients were placed in one group, while 30 patients with mitral restenosis, after successful initial percutaneous mitral valvuloplasty, were placed in another group. Ante grade trans-septal approach was adopted to perform percutaneous mitral valvuloplasty. The procedure was considered successful in achieving a 50% increase in the area of the mitral valve, without any major complication.

**Results:**

Procedural success in first PMV patients was more (18 patients; 90.0%) than in redo PMV patients (26 patients; 86.6%) (Non-significant). The patients in both groups didn’t differ significantly in terms of MVA after the procedure, the increase of MVA, the average difference in blood pressure across the mitral valve, and the complications experienced after the complete procedure. However, the final mitral valve area was negatively correlated with the initial area in both groups.

**Conclusion:**

Redo PMV for MR when performed after successful initial PMV is effective, has considerable rate of procedural success, which is achieved with a complication rate less as compared to initial PMV for de novo mitral stenosis.

## INTRODUCTION

Rheumatic valvular disease, primarily mitral valve stenosis (MS), is one of the most prevalent valve disorder in majority of the developing countries.^1^ In the past two decades, percutaneous mitral valvuloplasty (PMV) has been the most preferred treatment for symptomatic MS patients.^2^ This surgical technique has proved to be successful in both short and long term, and that too with the low risk of complications.^3^ Previously, randomized clinical studies have been carried out to compare the efficacy of percutaneous mitral valvuloplasty with commissurotomy, both open and closed, in patients with mitral valve morphology appropriate for balloon valvuloplasty. The result of these studies reported the dominance of PMV in better haemodynamic, echocardiographic, and clinical outcomes over other therapeutic strategy, and also to the brief hospital stay and lesser mortality and morbidity rates in case of PMV.^4^

The delayed recurrence of symptoms following percutaneous mitral valvuloplasty is exclusively linked with mitral restenosis. According to research, the rate of restenosis can be up to 40% at seven years follow up, but it can vary depending upon the centers and technique used. Not many studies have been conducted about redo PMV, but it has been observed that it showed promising results with favorable valve characteristics.^5^ Our study aims to evaluate and compare the effects of redo PMV with initial percutaneous mitral valvuloplasty in MR and de novo mitral stenosis patients, respectively.

## METHODS

A retrospective study was conducted at cardiology department of Ch.Pervaiz Elahi Institute of Cardiology Multan for the period of 1 year from 6^th^ July 2020 to 6^th^ July 2021. Fifty patients were included in the study. Initial 20 consecutive patients with symptomatic moderate to severe MS were added in the first PMV group if their mitral valve area (MVA) was < 1.5 cm^2^ and later 30 patients with mitral restenosis after successful PMV were added in the redo PMV group if the patients’ mitral valve area was > 1.5 cm^2^ with loss of more than 50% of the valve area gained initially by the 1^st^ PMV). The patients who were found to have mitral regurgitation limited to grade 2/4 on echocardiography were considered eligible for participation in the study. However, the patients with unfavorable mitral valve morphology (defined as a Massachusetts General Hospital [MGH] score equal to or less than 12), previous percutaneous mitral commissurotomy (PTMC), coexisting valve disease that requires surgical treatment, patients who were advised bypass surgery, and comorbid patients(like cancer) with risk of mortality were excluded from the study. Ethical Review Committee of the Hospital reviewed and approved the study with ref no.147-88 on dated 23-06-2020. Within 24 hours of the admission, transthoracic echocardiography was used to analyze the mitral valve of all patients. All the patients then underwent doppler echocardiography. Doppler spectral analysis was done to measure mean diastolic pressure across the mitral valve. Helmcke classification was used for colored flow mapping of MR jet in order to quantify MR grade.^6^ MGH scoring system was to score the mitral valve.^7^ The left atrium diameter was assessed in parasternal long (PLAX) and short-axis (PSAX) views. The sizes of other chambers and valve abnormalities were also evaluated. The trans-esophageal echocardiographic assessment was done on all patients just before the procedure using the same echocardiography machine. The patients went through a standard assessment to ascertain the MAD, septum premium thickness, and to eliminate the possibility of thrombi in the left atrium or left atrial appendage. Ante-grade trans-septal approach was used, by adopting either the standard balloon technique or the multitrack technique, to perform percutaneous mitral valvuloplasty. In the standard balloon technique, the balloon size was kept as described by Inoue et al.^8^ For the multitrack technique^9^, the size of the balloon was selected according to the mitral annulus diameter (MAD) (measured by ECG) such that the mitral diameter equates the diameter of the two balloons. Haemodynamic monitoring was done both prior to the procedure and following the procedure. The results were calculated by invasive measurement of the mean diastolic pressure gradient across the mitral valve. Following the procedure, patients were analyzed by TTE assessment after 48 hours to note the mitral valve area (by planimetry method), the mean diastolic pressure gradient across the mitral valve, and the presence and grade of MR, if any.

If a 50% or more increase was observed in the MVA such that the area after the procedure was greater than or equal to 1.5 cm^2^ without any severe complications, the procedure was regarded as a success. If any of the patients suffered cardiac tamponade, cerebrovascular stroke, per procedural death, or had more than grade 2/4 MR, it was regarded as a serious complication. If the mitral valve lost more than 50% of its initial gain area obtained by previous PMV, it is regarded as restenosis given that the final MVA is less than 1.5 cm^2^.

All normally distributed continuous variables were expressed as standard deviation (σ). Kolmogorov-Smirnov test was opted to evaluate the Gaussian distribution. To compare the distribution of categorical and continuous variables between the two groups, chi-square and unpaired t-tests were opted respectively. To study the relationship between the final MVA and the initial MGH score of the mitral valve in both groups, Pearson product-moment correlation coefficient was used. Two-tailed tests were performed, and the score was considered statistically significant if the value of p was < 0.05. The data analysis in the study was done by using the SPSS version 20.

## RESULTS

The average age of the study patients was 32.5 ± 5 years, among which 12 (24.0%) were male. Twelve patients (24.0%) were in atrial fibrillation (AF). Among the patients who underwent redo procedure after initial PMV, the average time between both procedures was 5.5 ± 2.0 years. There were no symptoms of rheumatic activity in any patients between both procedures. The patients’ baseline characteristics and initial observations after the ECG are shown in Table-I. The results showed no significant change between both groups except atrial fibrillation, which was seen more in the patients of redo PMV group. However, the difference remained insignificant.

**Table I:** Baseline clinical findings and initial echocardiographic characteristics in two study groups (N=50).

Variables	First PMV (n=20)	Redo PMV (n=30)	P-value
Age (years)	31.7± 5.7	33.5±6.2	NS
Males	4(20.0%)	8(26.6%)	NS
Atrial fibrillation	2(10.0%)	10(33.3%)	NS
NYHA class			NS
I	1(5.0%)	0(0%)	NS
II	14(70.0%)	24(80.0%)	NS
III	4(20.0%)	4(13.3%)	NS
IV	0(0%)	0(0%)	NS
Initial MVA (cm^2^)	1.0±0. 1	0.9±0.1	NS
Mean DPG (mmHg)	14± 5	14±4	NS
Peak DPG (mmHg)	21±7	20±8	NS
Total MGH score	6.6±1.1	6.6±1.5	NS
Thickness	1.9±0.1	1.9±0.4	NS
Motion restriction	1.9±0.4	1.8±0.3	NS
Calcification	1.8±0.2	1.9±0.4	NS
Subvalvular involvement	1.7±0.2	1.7±0.3	NS

(NS= non-significant).

In the first PMV group, 15 (75.0%) and 5 (25.0%) patients underwent the multitrack procedure and standard balloon technique, respectively. In the redo PMV group, 18 (60.0%) and 12 (40.0%) underwent multitrack technique, and the standard balloon technique, respectively (Non-Significant, NS). As per the definition of procedural success in our study, the procedure was successful in 18 (90.0%) and 26 (86.6%), respectively (NS). Hemodynamic and echocardiographic data of patients of both groups after the procedure are sown in Table-II. Both the groups did not differ significantly regarding any of the immediate procedural outcome variables.

**Table II:** Post-procedural hemodynamics and echocardiographic findings in two study groups (N=50).

Variables	First PMV (n=20)	Redo PMV (n=30)	P-value
***Doppler measurement***			
First MVA (cm2)	1.8±0.3	1.6±0.2	NS
Gain MVA (cm2)	0.6±0.2	0.6±0.1	NS
Mean DPG (mmHg)	5±1	5±2	NS
Peak DPG (mmHg)	9±2	10±2	NS
***Invasive measurement***			
Mean DPG (mmHg)	5±2	6±2	NS
Peak DPG (mmHg)	11±2	11±3	NS

In the first PMV group, one (5.0%) patient suffered a cerebrovascular stroke soon after the procedure and couldn’t survive. The patient had atrial fibrillation, but TEE revealed no thrombi the day prior to the procedure. He was administered the standard heparin dose after trans-septal puncture (5,000 IU), had a 45-minute long procedure, which is considered normal. Following the procedure, one (5.0%) patient had a suboptimal final MVA, one suffered haematoma formation at the puncture site, and one suffered rapid arterial fibrillation (AF) that was treated with electrical cardioversion.

In the redo PMV group, one (3.33%) patient suffered severe mitral regurgitation, one of them had grade 4/4 MR and underwent immediate mitral valve replacement. In contrast, the other had grade 3/4 MR and was treated without any invasive procedures. In this group, too, one (3.33%) patient had a suboptimal final MVA following the procedure. In addition, one patient suffered V-tach that required electrical cardioversion, one suffered a drug allergy, and another suffered a vasovagal syncope. None of the patients had cardiac tamponade or transfusion of blood needed or peripheral vascular repair. The mitral valve area after the procedure (correlation coefficient r = –0.439) and the initial MGH score of the mitral valve (correlation coefficient r = –0.387) in the two study groups were negatively related (p less than 0.05 for both).

## DISCUSSION

In this study, patients with de novo MS were compared with patients with mitral restenosis after successful initial percutaneous mitral valvuloplasty. Since much work hasn’t been conducted on evaluated topic in Pakistan, a considerable procedural success rate found in our study provides useful insights in evaluated disorder. The redo PMV showed 86.6% procedural success with a low complication rate as compared first PMV.

Valve restenosis is the primary cause of functional decline following a successful PTMC.^10^ The incidence of restenosis varies in research, but the recurrence rate is 7–21% following successful PMV.^11^ PMV has recently been introduced as a treatment for symptomatic mitral restenosis and has shown favorable mid-term results, especially in patients with good valve traits.

The immediate procedural success rate was also more in the redo procedure than initial PMV (86.6% vs. 90.0%, respectively). These results are similar to results in Jung et al.^12^ and better than those recorded by Pathan et al. (91% and 75%, respectively). The low procedural success in Pathan et al.^13^ may be due to old age, high valve calcification, more atrial fibrillation, higher MGH score older age, and history of mitral valve repair surgery. Young age, like in our study, 32.5 ± 5 years, contributes significantly to procedural success. In addition, atrial fibrillation is a sign of poor outcome. In the present study, more patients in the redo group had AF (p less than 0.05), due to which there was a high rate (3.33%) of mitral regurgitation in this group. However, the procedural success did not differ significantly in both groups. Therefore, similar patient characteristics may have improved the results of the redo procedure.

A lot of research has been done to demonstrate the safety of PMV. In our study, only 3.33% of patients developed severe MR, and there was no case in the first PMV group. It is not easy to compare the results of both groups due to differences in patients’ characteristics, recurrent valve injury, and the mitigation process depending upon various factors. The complications in this procedure are unpredictable as only one patient (5%) suffered a stroke. Though the patient had atrial fibrillation with no thrombi as indicated by ECG, his valve calcification score was 3/4. This justifies that only symptomatic patients may preferably undergo redo PMV.

The patients with MGH scores equal to or greater than 12 were excluded from our study, and this score was not significant between the study groups. The favorable mitral valve morphology in our study can also be a factor contributing to high procedural success. According to recent studies, it is revealed that commissural morphology, especially commissural calcification and subvalvular involvement, influences the final results of PMV. Therefore, a new scoring system other than the standard MGH score was suggested that focused on these two variables and predicted the procedure outcome more accurately. If factors unrelated to the outcome were excluded (i.e., leaflet thickness and mobility), a better procedural success rate would have been obtained in our study (86.6%).

It has been observed that the underlying disease continues to progress even after successful mitral valve dilation. A recent study^14^ has addressed the increase of left atrial volume progressively despite successful PMV. This has been related to the initial atrial fibrillation, a higher MGH score, less mitral valve area after the procedure, and a greater immediate post-procedural left atrial volume index. This indicates that chronic pressure overload on the left atrium is not only a sign of left atrial enlargement (LAE) but can also cause structural and ultrastructural changes, like myocardial hypertrophy, Interstitial lung disease (ILD) and myocardial cell loss. Not much data has been reported about mitral regurgitation after the procedure, but two articles^15^,^16^ have reported the regression of MR severity at long-term follow-up. MVA is mainly increased by commissural splitting during percutaneous mitral vulvoplasty. Therefore, the regression of MR after PMV at long-term follow-up may be due to gradual healing of over-split commissures and/or restoration of papillary muscle function. In our study, the factors of MGH score (i.e., motion, subvalvular involvement, mitral valve leaflet thickness, and calcification) did not differ significantly between the patient groups. They thus may have led to a similar procedural success score.

A 5-year study^17^ showed good long-term follow--up outcomes of redo PMV. The restenosis-free survival was 95 ± 1%, and event-free survival was 94.5 ± 1.3%. In addition, the MVA was more than 1.8 cm^2^ after the procedure, which indicated restenosis as well as event-free survival rates at 5-year follow-up. The follow-up data of our study is also consistent with the results of this study.

As redo PMV results in a high procedural success rate and a low complication rate compared to the first PMV for de novo MS, it is a more promising treatment of recurrent MS. In contrast to surgery, it is a more appealing long-term treatment option. It is more favorable for young fertile women to avoid valve replacement. Additionally, patients with the risk of post-operative mortality can also prefer this procedure over surgery.

### Limitations of the study

It is a single center study with fewer study subjects, limited age range, only tested using two techniques, making our results less general for all MS patients. A multi-center study with more patients may yield better and generalized results. The procedural success was on the basis of data obtained from ECG, not taking into account hemodynamic data. Therefore, the intermediate and long-term outcomes can only be determined by the comparative follow-up of both groups.

## CONCLUSION

Redo PMV for MS when performed after successful initial PMV is feasible, has considerable rate of procedural success, which is achieved with a reduced complication rate as compared to initial PMV for de novo mitral stenosis.

### Authors’ Contribution:

**MR, MKJ, J:** Conceived, designed, did statistical analysis & editing of manuscript.

**HMR, Kashif:** Did data collection and manuscript writing.

**MR, MKJ, HMR:** Were involved in detailed review and final approval of manuscript.
